# Increased Bilateral Interactions in Middle-Aged Subjects

**DOI:** 10.3389/fnagi.2014.00005

**Published:** 2014-01-24

**Authors:** Jolien Heetkamp, Tibor Hortobágyi, Inge Zijdewind

**Affiliations:** ^1^Department of Neuroscience, University Medical Center Groningen, University of Groningen, Groningen, Netherlands; ^2^Center for Human Movement Sciences, University Medical Center Groningen, University of Groningen, Groningen, Netherlands; ^3^Faculty of Health and Life Sciences, Northumbria University, Newcastle Upon Tyne, UK

**Keywords:** fatigue, associated muscle activity, middle-aged, bimanual interactions, ipsilateral corticospinal excitability

## Abstract

A hallmark of the age-related neural reorganization is that old versus young adults execute typical motor tasks by a more diffuse neural activation pattern including stronger ipsilateral activation during unilateral tasks. Whether such changes in neural activation are present already at middle age and affect bimanual interactions is unknown. We compared the amount of associated activity, i.e., muscle activity and force produced by the non-task hand and motor evoked potentials (MEPs) produced by magnetic brain stimulation between young (mean 24 years, *n* = 10) and middle-aged (mean 50 years, *n* = 10) subjects during brief unilateral (seven levels of % maximal voluntary contractions, MVCs) and bilateral contractions (4 × 7 levels of % MVC combinations), and during a 120-s-long MVC of sustained unilateral index finger abduction. During the force production, the excitability of the ipsilateral (iM1) or contralateral primary motor cortex (cM1) was assessed. The associated activity in the “resting” hand was ~2-fold higher in middle-aged (28% of MVC) versus young adults (11% of MVC) during brief unilateral MVCs. After controlling for the background muscle activity, MEPs in iM1 were similar in the two groups during brief unilateral contractions. Only at low (bilateral) forces, MEPs evoked in cM1 were 30% higher in the middle-aged versus young adults. At the start of the sustained contraction, the associated activity was higher in the middle-aged versus young subjects and increased progressively in both groups (30 versus 15% MVC at 120 s, respectively). MEPs were greater at the start of the sustained contraction in middle-aged subjects but increased further during the contraction only in young adults. Under these experimental conditions, the data provide evidence for the reorganization of neural control of unilateral force production as early as age 50. Future studies will determine if the altered neural control of such inter-manual interactions are of functional significance.

## Introduction

The contralateral primary motor cortex (M1) is the main controller of unimanual voluntary movements. However, evidence shows that especially when a task is more complex or requires a strong effort, the ipsilateral M1 (iM1) becomes increasingly active (Hess et al., 1986; Ugawa et al., 1993; Stedman et al., 1998; Tinazzi and Zanette, 1998; Hortobagyi et al., 2003; Zijdewind et al., 2006; van Duinen et al., 2007; Perez and Cohen, 2009; Post et al., 2009b). These excitatory ipsilateral effects can be so strong that contralateral, homologous muscles become active (Curshmann, 1906; Cernacek, 1961; Armatas et al., 1994; Zijdewind and Kernell, 2001; Cincotta and Ziemann, 2008; Post et al., 2009b). Compared with isolated strong muscle contractions, activation of ipsilateral cortical motor areas is even more pronounced during a sustained fatiguing contraction (Liu et al., 2003; Post et al., 2009b) and is accompanied by increased levels of associated contralateral muscle activity (Zijdewind and Kernell, 2001; Post et al., 2009a). This associated activity results in behavioral changes because subjects start to perform overt, associated movements with the hand contralateral to the target hand.

With advancing age, ipsilateral motor areas – including iM1 – become functionally more relevant (Talelli et al., 2008a; Fling et al., 2011; Boudrias et al., 2012; Fling and Seidler, 2012). One prediction could be that performing a unimanual sustained voluntary contraction would generate higher levels of excitability in the iM1 of old when compared with young adults, resulting in a greater contralateral associated activity. Because imaging studies across the lifespan suggest the appearance of anatomical changes in the motor brain already in late midlife (Haug and Eggers, 1991; Marner et al., 2003; Madden et al., 2004; Seidler et al., 2010; Taki et al., 2013; Zhou et al., 2013), it is reasonable to expect that neurophysiological changes in M1 and iM1 are also present. Therefore, the purpose of the present study was to determine if middle-aged compared with young adults execute unilateral motor tasks with higher iM1 excitability and with higher levels of associated contralateral activity. An additional question is whether a sustained contraction would further increase the already elevated ipsilateral activation and associated activity in middle-aged subjects. In other words, would middle-aged subjects who already demonstrate associated activity at the start of the contraction increase this associated activity even more during a fatiguing contraction or do they just start at higher levels and end up producing the same amount of associated activity as young subjects? Data in older adults (>65 years) provide evidence for this latter scenario (Shinohara et al., 2003). However, if increased cortical activation is required to maintain appropriate voluntary drive to the relevant muscles, as is suggested by data of McNeil et al. (2009, 2011a), one would expect that the increase would be similar in all participants, independent of the starting point. A decrease or a smaller increase in cortical activation would otherwise result in diminished activation of the target muscles. Thus, if ipsilateral activation in the middle-aged subjects reflects the amount of effort added upon increased ipsilateral activation (due to age-related changes), one would expect a progressive increase in associated activity during a sustained contraction.

## Materials and Methods

### Subjects

**Figure 1:**
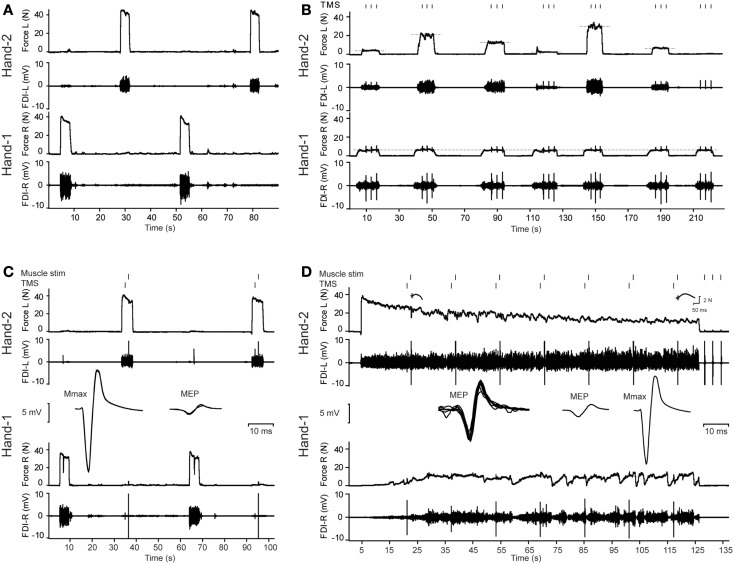
**Overview of the protocol and illustrative data from a middle-aged male subject**. **(A)** Three maximal voluntary contractions (MVCs) of the first dorsal interosseus (FDI) intermittently with the left and right hand (only two MVCs are presented in this figure). **(B)** Example of the bilateral contractions. The subject started with Hand-1 (in this figure the right hand) followed by Hand-2 (in this figure the left hand). During the contractions, three transcranial magnetic stimuli (TMS) were given to the motor cortex contralateral to Hand-1. Feedback of the target and actual produced forces were presented to the subject on-line. **(C)** MVCs combined with muscle stimulation of Hand-2 and TMS of the motor cortex contralateral to Hand-1. Before the MVCs, the ulnar nerve of Hand-1 was stimulated to evoke maximal *M*-waves (*M*_max_). **(C)** Shows an overlay of five *M*_max_ responses, followed by an overlay of three MEPs (recorded in the right hand) obtained during MVCs of the left hand. **(D)** Two-minute contraction with Hand-2. During the contractions, MEPs were evoked in Hand-1; an overlay of all MEPs during the fatigue test is presented together with the MEP during a brief contraction of the left hand and *M*_max_ (*n* = 5) obtained after the fatiguing task. Note the increase in associated activity in Hand-1.

Twenty right-handed volunteers (10 females) were subdivided into two age groups, young adults (*n* = 10, age 24 years, 2 SD, range 21–27; 5 females) and middle-aged adults (*n* = 10, age 50 years, 3 SD, range 46–53, 5 females). Prior to the experiment, a checklist was issued to exclude subjects with migraine, neurologic or muscle diseases, or epileptic episodes. All subjects signed an informed consent, approved by the local ethics committee. Right handedness was confirmed with the Handedness Questionnaire (Brainmapping.org. adapted from Oldfield, 1971; mean laterality index: 89.8, range 65–100). All subjects performed the experiment twice, 1 week apart. In one experiment, transcranial magnetic brain stimulation (TMS) targeted the right hand and in the other experiment, the left hand. The order of the experiments was alternated between subjects. In the methods, we describe the set-up for the experiment in which TMS was given to the left motor cortex (see Figure 1).

### Experimental set-up

Subjects sat in a height-adjustable chair with both arms resting on a table instrumented with force transducers. With the elbows flexed at 135°, both forearms were clamped in a vertical position halfway between pronation and supination. The wrists and hands were kept in a vertical position with pressure plates. The middle, ring, and little fingers were mechanically isolated from the index finger using a small plastic plate. The thumb was slightly stretched toward extension with a strap. Both index fingers were inserted into snugly fitting rings around the proximal interphalangeal joint, and connected to a force transducer (Zijdewind and Kernell, 1994), which was adjustable in height to keep the index fingers in a slightly abducted position.

#### EMG recordings

Surface electromyographic (EMG) activity was recorded from the first dorsal interosseus (FDI) muscle of both hands. For the right FDI, one electrode (Ag–AgCl, 4 mm, *in vivo* metrics, Gainesville, FL, USA) was placed over the muscle belly and a second electrode close to on the metacarpophalangeal joint of the index finger. For the left FDI, one adhesive electrode was placed on the proximal border of FDI muscle, in between the first and second metacarpal bones. The second electrode was placed on the distal border of the FDI (see Zijdewind et al., 1998). This set of electrodes was also used to electrically stimulate the left FDI muscle (see “Muscle stimulation”). Adhesive grounding electrodes were strapped around each wrist.

Force and EMG data were amplified and sampled at 2000, 5000, and 500 Hz for EMG, motor evoked potentials (MEPs), and force recordings, respectively (CED 1401 plus interface, Cambridge Electronic Design, UK). Data were stored on a personal computer for off-line analysis.

### Stimulation

#### Electrical nerve stimulation

Adhesive electrodes were placed on the skin at the wrist above the ulnar nerve. The ulnar nerve of the right side was stimulated with increasing current (5 mA steps, Digitimer DS7, UK) to obtain maximal *M*-waves (*M*_max_). During the experiment, the ulnar nerve was stimulated at 130% of the intensity to obtain *M*_max_.

#### Muscle stimulation

To estimate voluntary drive, we superimposed twitches on maximal voluntary contractions (MVCs) of the left FDI (twitch superimposition technique, Merton, 1954; Allen et al., 1995; Zijdewind et al., 1998). The stimulation consisted of two 200-μs-long pulses with an inter-pulse interval of 10 ms. We used the same electrodes for EMG recording and electrical stimulation of the left FDI (see “EMG Recordings”). The stimulating current was increased in steps of 5 mA until maximal force was evoked. Throughout the experiment, the current that produced the maximal twitch force was used for the muscle stimulation. The force evoked by the paired-pulse stimulation is referred to as doublet force.

#### Transcranial magnetic stimulation

A figure-of-eight branding iron coil (diameter 50 mm) connected to a TMS stimulator (Magstim 200 rapid, Dyfed, UK) was used to elicit MEPs in the contralateral FDI muscle. Subjects wore a tight fitting cap and the location on the head where the biggest response in the contralateral FDI could be evoked was marked. Because the rapid stimulator produces a biphasic pulse, both coil current directions were examined in a subject as suggested by the manufacturer. In all subjects and in both hemispheres, lower stimulation thresholds were found with the current in the coil in an anterio-posterior position. Resting motor threshold (RMT) was defined as the lowest stimulation intensity required for eliciting MEPs in the contralateral FDI muscle at rest of at least 50 μV in at least three out of five consecutive trials (Rossini et al., 1994). During the experiment, 110% of the RMT was used.

### Experimental tasks

The experiment was conducted twice in each subject with at least 1 week apart, preferably on the same day of the week but always at the same time. If during the first experiment MEPs and *M*-waves were evoked in the right FDI and superimposed twitches in the left FDI, then in the second experiment MEPs and *M*-waves were evoked in the left FDI and superimposed twitches in the right FDI. Half of the subjects started with TMS and *M*-waves in the right FDI, the other half with the left FDI.

The experiment consisted of four tasks. Continuous feedback of the index finger abduction force produced by the left and right FDI with the lines for the target forces being displayed in real-time on a computer screen in front of the subject (see Figure 1).

#### Maximal voluntary contractions

Subjects produced three MVCs with the left and right FDI with 1-min of rest between consecutive contractions, alternating between the two sides (Figure 1A). During the MVCs, maximal force and RMS-EMG (100 ms around the maximal peak force) was determined during each contraction, as well as the maximal force and RMS-EMG (100 ms around peak force) produced by the contralateral FDI.

#### Unilateral and bilateral sub-maximal contractions with MEPs

After the MVCs, subjects produced an abduction force with their right index finger (Hand-1) at four force levels (0, 5, 15, or 30% of MVC). When the subject reached the required force level, they were requested to produce a concurrent force with their left index finger abductor (Hand-2). Subjects received continuous feedback of the target and the produced forces.

During a set of contractions, the force level of Hand-1 was fixed at one of the four levels, whereas the force levels of Hand-2 was varied between 0, 5, 10, 15, 30, 50, and 70% of MVC (Figure 1B). Subjects were instructed to maintain the required force levels until the delivery of three TMS pulses was complete. A contraction lasted about 20 s and the subject received at least 30 s of rest between the contractions. One set of seven contractions lasted about 6 min.

In total, all subjects performed unilateral contractions with the FDI of Hand-1 (5, 15, or 30% of MVC) and Hand-2 (5, 10, 15, 30, 50, and 70% of MVC) and bilateral contractions consisting of 18 force combinations (Hand-1: 5, 15, or 30% of MVC and Hand-2: 5, 10, 15, 30, 50, and 70% of MVC). The MEPs were always evoked in the FDI of Hand-1, i.e., the hand that started the bilateral contraction.

#### Maximal voluntary contraction with twitch interpolation

After finishing the sub-maximal force task, we repeated the MVC measurements combined with different forms of stimulation (Figure 1C). During the MVCs of Hand-1 (right hand), the subject received a TMS stimulus over the (left) motor cortex contralateral to the contracting FDI. During MVCs with Hand-2 (left hand), a TMS stimulus was given over the (left) motor cortex ipsilateral to the contracting FDI, followed by an electrical stimulus to the contracting (left) FDI muscle (twitch superimposition; inter-stimulus interval between TMS pulse and electrical simulation, 1.5 s).

#### Sustained, fatiguing maximal contraction

Subjects produced a 2-min sustained contraction with the FDI of Hand-2 (left hand, i.e., the FDI muscle that received the muscle stimulation). The instruction to the subjects was to produce maximal abduction force with the left index finger for 2 min; no further instructions were given regarding their contralateral hand. Subjects were verbally encouraged to give maximum effort throughout the sustained contraction. During the 2 min, TMS was applied seven times to the (left) hemisphere ipsilateral to the fatiguing muscle (i.e., the Hand-2 FDI) followed by electrical muscle stimulation of the (left) fatiguing FDI (i.e., Hand-2 FDI). The interval between each pair of stimulating pulses (the TMS and electrical muscle stimulation) was 17 s and the interval between TMS and muscle stimulation was 1.5 s (Figure 1D). After 2 min, subjects relaxed the index finger abductor and muscle stimulation was applied three times within 5 s after the end of the fatiguing contraction, which was immediately followed by a brief MVC with the Hand-1 FDI (right non-fatigued FDI) during which TMS was applied and a brief MVC with Hand-2 FDI (left fatigued FDI) during which TMS and muscle stimulation was applied. To finish the protocol, ulnar nerve stimulation was applied five times in the Hand-1 FDI (right hand).

In the second experiment, the TMS and muscle stimulation were delivered to the contralateral side. The fatiguing task was also performed with the contralateral muscle.

### Data analysis

#### Maximal voluntary contractions

During the MVCs, the maximal force and RMS-EMG (100 ms time window around peak force) was determined. The maximal force value during the MVCs was considered as the “true” MVC and force values obtained during voluntary and associated contractions were expressed as a percentage of this MVC value. The maximal RMS-EMG value obtained during MVCs was used to normalize all EMG values (both voluntary contractions and associated activity). The RMS-EMG and force values during the associated contractions of the contralateral FDI during the MVCs were averaged over the three MVC-trials.

The MVC and associated activity was analyzed with repeated measures mixed design ANOVA with Hand (left and right) as within-subject factors, and Age-group (young and middle-aged) and sex (male and female) as between-subject factors.

#### Unilateral and bilateral sub-maximal contractions

When the subjects reached their force targets, the mean index finger abduction force was measured for a 1-s time window before the TMS stimulus. The three 1-s values obtained during one contraction were averaged. For the same time window, the mean RMS-EMG values were determined for both FDI muscles. Additionally, the mean RMS-EMG in the Hand-1 FDI was assessed 100 ms before the TMS stimulus. All force and EMG values were expressed as percentage of the MVC values. The amplitude and area of the MEPs were expressed as a percentage of the *M*-wave. To quantify performance on the bilateral task, we calculated the force difference with the following Eq. (1):

$$\begin{array}{llll} & \mathrm{Bilateral force difference}\; & & \;\\ & \;=\sqrt{\begin{array}{c} \left({\left({\mathrm{target force}}_{\mathrm{Hand-1}}-{\mathrm{produced force}}_{\mathrm{Hand-1}}\right)}^{2}\right.\\ {\left.\;+\left({\mathrm{target force}}_{\mathrm{Hand-2}}-{\mathrm{produced force}}_{\mathrm{Hand-2}}\right)\right)}^{2} \end{array}}\; & & \; \end{array}$$

To investigate the effect of a contraction on the ipsilateral motor cortex excitability, we choose to analyze the force, MEP, and EMG values obtained during unilateral and bilateral contractions separately. During the unilateral contractions, changes in force, MEP amplitude and area, and background RMS-EMG were statistically analyzed with a repeated measures mixed design ANOVA. As within-subject factors were identified, force of Hand-1 (Force-H1: 5, 15, and 30% of MVC) or force of Hand-2 (Force-H2: 5, 10, 15, 30, 50, and 70% of MVC) and Hand (left and right); Age-group (young and middle-aged) was assigned as a between-subject factors.

During the bilateral contractions, we analyzed force difference, MEP, and EMG values. The mixed design repeated measures ANOVA that was used consisted of Force-H1 (5, 15, and 30% of MVC), Force-H2 (5, 10, 15, 30, 50, and 70% of MVC) and Hand (left and right) as within-subject factors, and Age-group (young and middle-aged) as a between-subject factors. For unilateral and bilateral contractions, *post hoc* analysis (least significant difference, LSD) in which we compared the data obtained at 5% of MVC versus data obtained at higher force levels (for Hand-1: 15 and 30% of MVC, and Hand-2: 10, 15, 30, 50, and 70% of MVC) were performed to break down significant effects where appropriate.

#### Fatigue task

Mean force and RMS-EMG values were obtained for 2-s time periods during the sustained contraction. Epochs in which TMS stimuli or muscle stimuli were given (7 × 4 s-periods) were excluded from the analysis. This resulted in 46 epochs of 2 s. The mean force and RMS-EMG data were expressed as a percentage of the pre-fatigue MVC values. The forces evoked by the muscle stimulation were measured and expressed as a percentage of the potentiated doublet-force obtained before the fatiguing contractions. The amplitude and area of the MEP was measured and expressed as a percentage of the *M*-wave.

Changes in force and EMG values (% MVC) during the sustained maximal contraction were analyzed with a repeated measures mixed design ANOVA (2 × 46 × 2) with Hand (left and right) and Time (1–46 periods of 2 s) as within-subjects factors; Age-group was included as a between-subject factor.

Some of the doublet-forces evoked during the sustained contraction could not be analyzed due to fast variations in voluntary background force. It was then impossible to decide which part of the force increase was due to voluntary activation and which part was due to the evoked twitch. Instead of using a fixed time interval to determine the evoked force, we excluded this data point and analyzed the twitch forces with a multilevel analysis. The multilevel analysis is less affected by missing data points (in contrast to the repeated measures ANOVA; Snijders and Bosker, 1999). We also used the multilevel analysis for the MEPs evoked during unilateral contractions of Hand-2. During these contractions (especially at higher forces), some subjects could not completely relax Hand-1 and EMG activity was visible before the TMS stimulus. These MEPs were excluded from the analysis. Because this resulted in missing data points in some subjects, we choose to perform multilevel statistical tests.

Associations between the relevant parameters (MVC, amount of associated activity, fatigability) obtained in the two sessions were determined with linear regression analysis. Similar statistics were used to explore associations between values of associated activity and bimanual interference.

Statistical significance was set at α = 0.05; if violation of sphericity of the data occurred, degrees of freedom were corrected using Huynh–Feldt estimates of sphericity.

## Results

Table 1 shows the main characteristics of the subjects. There was no difference in the intensity used for muscle, nerve, and cortical stimulation between the two experiments and age-groups (*p* > 0.1). During the sustained contraction, the force recording in one subject and the EMG recording in another subject could not be used due to technical problems. There was some noise interference (>10 μV) in 20% of the EMG recordings. This noise could be due to having both the electrical stimulator and the EMG amplifier simultaneously connected to the electrodes. Although the noise was small, the noise occurred more often in the middle-aged subjects than in the younger subjects. Therefore, we analyzed in those cases, both the non-filtered and notch-filtered EMG signals (IRR 50 Hz filter). In the text, we present the notch-filtered EMG data, if the analysis shows differences between the non-filtered and notch-filtered data, we present both results.

**Table 1:** **Subject characteristics displayed in mean ± SD**.

	Young adults (*n* = 10)	Middle-aged adults (*n* = 10)	Total
Age (years)	23.6 (2.3)	49.8 (2.5)	36.7 (13.6)
Laterality index	86.5 (14.3)	93.0 (10.9)	89.8 (12.8)
RMT (% max stimulator output)	55.1 (6.6)	59.5 (8.5)	57.5 (7.9)
Muscle stimulation (mA)	36.2 (9.9)	38.7 (9.1)	37.6 (9.4)
Nerve stimulation (mA)	36.1 (9.8)	41.8 (8.7)	38.7 (9.1)
*M*-wave (mV)	24.5 (6.0)	24.6 (5.7)	24.5 (5.3)
Rest MEP^a^ (mV)	0.6 (0.4)	0.7 (0.5)	0.7 (0.4)

*Age in years, laterality index, RMT in percentage of maximum stimulator output, muscle and nerve stimulation in mA*.

*^a^ Rest MEP at 100% resting motor threshold*.

### Maximal voluntary contractions are similar in the two age groups

Males (45.2 *N*, 7.3 SD) had a higher maximal index finger abduction force than females [33.6 *N*, 6.8 SD; *F*_(1,16)_ = 19.10, *p* < 0.001]. The MVC force was not significantly different between the two age groups (young adults: 41.6 *N*, 8.7 SD; middle age adults: 37.2 *N*, 9.2 SD; *p* = 0.12), neither between the left (39.0 *N*, 9.0 SD) and right hand (39.7 *N*, 9.4 SD; *p* = 0.67), nor between the two experiments (*p* = 0.67). No interaction effects between Sex, Experiment, Age-group, or Hand were found (*p* > 0.25, for all interactions). For the MVC force in both the left and right hand, there was an association between the two experiments and there was an association between the MVC force of the left and right hand of the participants [*F*_(1,38)_ = 123.20, *R*^2^ = 0.76 and *F*_(1,38)_ = 39.10, *R*^2^ = 0.51, respectively; both *p* < 0.001].

### Amount of associated activity differs between the two age groups

During the MVCs, most subjects showed some amount of contralateral activity. Statistical analysis demonstrated main effects of Experiment [force: *F*_(1,16)_ = 5.04, *p* = 0.04] and Age-group [force: *F*_(1,16)_ = 4.43, *p* = 0.05; EMG: *F*_(1,16)_ = 5.23, *p* = 0.04]. In addition, there was an Age-group by Experiment by Sex interaction for both the associated force [*F*_(1,16)_ = 6.76, *p* = 0.02] and associated EMG activity [*F*_(1,16)_ = 4.58, *p* = 0.05]. Associated activity was higher in middle-aged subjects (force: 28.9 and 11.5% MVC, for middle-aged and young subjects, respectively), and higher in females (force: 26.7 and 18.7% MVC, EMG: 22.4 and 15.7% MVC; for Experiments 1 and 2, respectively) compared with males (force: 14.4 and 12.8% MVC, EMG: 11.7 and 11.1% MVC; for Experiments 1 and 2, respectively) in Experiment 1. The amount of associated activity in Experiments 1 and 2, and also in left and right hands were positively associated [*F*_(1,38)_ = 49.68, *R*^2^ = 0.57 and *F*_(1,38)_ = 47.33, *R*^2^ = 0.55, for EMG and force between the two experiments, respectively; *F*_(1,38)_ = 82.38, *R*^2^ = 0.68 and *F*_(1,38)_ = 67.48, *R*^2^ = 0.64, for EMG and force of the left and right hand, respectively; *p* < 0.001 in all cases].

### Unilateral contractions with TMS of the contralateral M1

To investigate possible differences between the age groups in the effect of muscle activation on the MEPs, participants performed unilateral contractions with their FDI (Hand-1) at three different force levels (5, 15, and 30% MVC) and were instructed to keep their contralateral FDI (Hand-2) relaxed. During the contraction, the contralateral motor cortex was stimulated three times (inter-stimulus interval >3 s).

**Figure 2:**
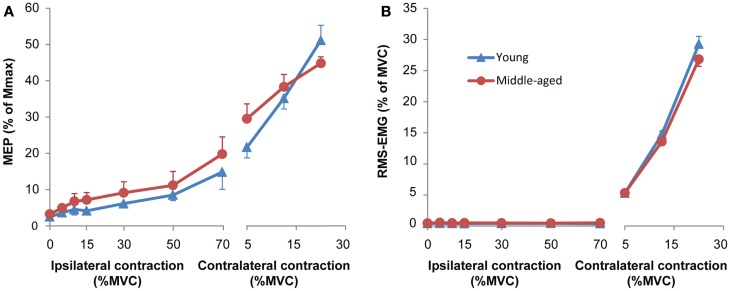
**(A)** Mean and standard error of motor evoked potentials (MEPs) during unilateral force production for young (blue) and middle-aged subjects (red). The left side of the panel shows MEPs obtained during an ipsilateral contraction at increasing force levels. Only MEPs were included that were not preceded by any voluntary activity; all MEPs of 8 out of 40 experiments (seven subjects: five middle-aged, two young adults) were excluded because more than half of the MEPs at high force levels showed small signs of EMG activity. The right side of the panel the MEPs obtained during a contralateral contraction (only for the MEPs of the same subjects as included in the ipsilateral contraction). The MEPs increased with ipsilateral and contralateral force without significant difference between young and middle-aged subjects. **(B)** Mean and standard error of RMS-EMG (100 ms before MEP) upon which the MEPs were evoked at increasing force levels of the hand ipsilateral to the motor cortex receiving the TMS pulse (left panel) and the contralateral hand (right panel) for young (blue) and middle-aged subjects (red). The background RMS-EMG was close to zero during the ipsilateral contraction and increased proportional with the force during the contralateral contraction. No differences were observed between young and middle-aged subjects.

All subjects performed the task as expected. The force data showed an Age-group by Hand by Force interaction for Force difference [*F*_(1.7,30.6)_ = 3.60, *p* = 0.05] because young subjects produced a marginally larger force with their left FDI at high force levels than middle-aged subjects (Force difference left FDI: young: +0.29% MVC and middle-aged: −1.3% MVC at 30% MVC target; *p* = 0.05). The evoked MEPs increased with increasing force and EMG levels [Figures 2A,B, right side; *F*_(2,36)_ = 36.76, *p* < 0.001; larger MEPs at 15% (*p* = 0.001) and 30% of MVC (*p* < 0.001)] without a difference between the two Age-groups (*p* = 0.49).

### Unilateral contractions with TMS of the ipsilateral M1

The purpose of these runs was to determine: (a) the age-related differences in iM1 activation and (b) the magnitude of associated force and EMG activity in the resting hand. Participants performed unilateral contractions with their FDI of Hand-2 (ipsilateral to the cortex receiving TMS) at six different force levels (5, 10, 15, 30, 50, and 70% of MVC). They were instructed to keep their contralateral hand relaxed (i.e., the hand in which MEPs were evoked). However, if subjects forcefully contracted the FDI of Hand-2, the FDI of Hand-1 also became activated [force: *F*_(1.2,21.5)_ = 4.37, *p* = 0.04; RMS-EMG: *F*_(1.3,23.1)_ = 5.20, *p* = 0.03, Figure 4A; larger force in Hand-1 when ipsilateral hand contracted at 50% (*p* = 0.02) and a trend toward significance at 70% of MVC (*p* = 0.06); larger RMS-EMG in Hand-1 when the ipsilateral hand contracted at 70% of MVC (*p* = 0.03)].

As expected from the changes in force and EMG in Hand-1, the MEPs evoked in the FDI of Hand-1 increased together with the activation of the FDI in Hand-2 [*F*_(2.4,43.9)_ = 34.73, *p* < 0.001; from 4.4% of *M*_max_ (0.6 SD) at 5% MVC force to 6.4 of *M*_max_ at 30% (*p* = 0.02), 9.8% of *M*_max_ (1.2 SD) at 50% MVC (*p* < 0.001), and to 17.5% of *M*_max_ (2.2 SD) at 70% MVC (*p* < 0.001)]. To correct for the increase in background EMG, we excluded all MEPs from the statistical analysis that were preceded by EMG (100 ms before the TMS stimulus). In eight experiments (seven subjects, five middle-aged subjects), no MEPs at force levels above 30% of MVC remained after rejection of trials with background EMG. We therefore excluded all of the data for these subjects from the statistical analyses. To be able to perform the analysis despite missing data points, we analyzed the MEPs with multilevel analysis. The analysis showed a relationship between the RMS-EMG in Hand-2 FDI and the MEPs [*F*_(1,31.14)_ = 31.14, *p* < 0.001; Figure 2A, left side], no main or interaction effect of Age-group was found.

### Bilateral contractions

The purpose of these runs was to determine the effects of age on bilateral interaction. We parametrically increased the contraction intensity (5, 10, 15, 30, 50, and 70% of MVC) in one FDI (Hand-2) and measured the corticospinal excitability in the other FDI while contracting at three low intensities (5, 15, or 30% of MVC; Hand-1, Figures 1 and 4). During these contractions, three magnetic stimuli were given to evoke MEPs in the FDI of Hand-1.

**Figure 3:**
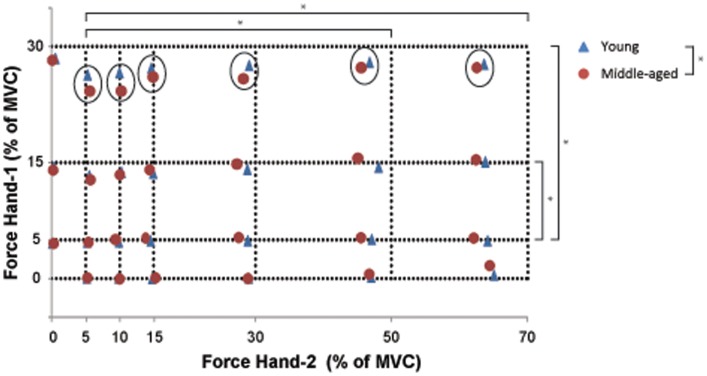
**The combination of bimanual forces produced by young (blue) and middle-aged subjects (red)**. The crossing of the interrupted lines represents the target force combination for Hand-1 (*y*-axis) and Hand-2 (*x*-axis). The measure of bilateral performance (force difference) is calculated as the Euclidean distance between the requested force combination and the produced force combination (see Eq. (1). Significant differences in force difference across bilateral force combinations are indicated by an asterisk (unilateral contractions were not included in the statistical analysis; main effect of Hand-1 and -2). The circles point to an Age-group effect on the amplitude of the force difference at high force levels in Hand-1. Overall, the force difference was larger for the middle-aged than for the young participants.

To quantify the performance in the bilateral task, we used Eq. (1) to calculate the difference between the target force and the force subjects actually produced. The force difference showed significant main effects for the force levels of Hand-1 [*F*_(2,34)_ = 26.68, *p* < 0.001; 5 versus 15%, *p* = 0.03, versus 30% *p* < 0.001], Hand-2 [*F*_(1.8,29.8)_ = 40.63, *p* < 0.01; 5 versus 50 and 70%, both *p* < 0.001], and an interaction effect for Hand-1 by Hand-2 [*F*_(6.4,108.3)_ = 4.14, *p* = 0.001; see Figure 3 for contrasts] with the force difference being larger at higher force levels of Hand-1 (Figure 3). There was an Age-group by Hand interaction in the force difference [*F*_(1,17)_ = 7.4, *p* = 0.01]; the force difference was smaller when the left hand started the contraction in the young subjects. Overall, the force difference was larger for middle-aged subjects than for young subjects [*F*_(1,17)_ = 4.56, *p* = 0.05].

**Figure 4:**
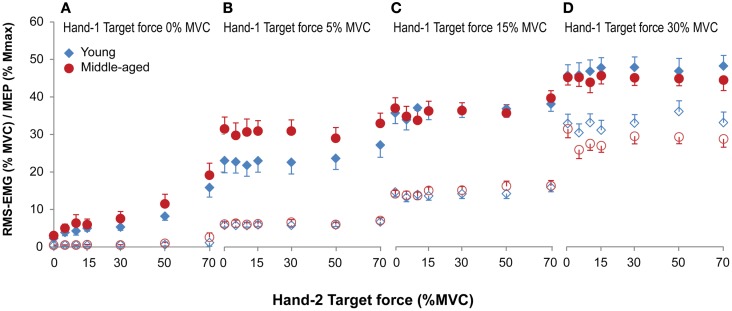
Mean and standard error of RMS-EMG (100 ms, open symbols, bottom row) and MEPs (closed symbols, top row) during unilateral **(A)** and bilateral **(B–D)** forces at different force combinations for young (blue) and middle-aged (red) subjects. Hand-1 is the hand in which the MEPs in the FDI were evoked and the hand that started the contraction during the bilateral contractions. For the values presented in **(A)** and the unilateral contractions (force Hand-2 equals 0) in **(B–D)** all MEPs were included for calculating the mean values. **(A)** Shows a small but significant increase in RMS-EMG and an increase in MEP amplitude with increasing ipsilateral force. **(B–D)** Shows that the RMS-EMG and the MEPs in Hand-1 were affected by both the force production of Hand-1 and -2. At low force levels (5% MVC) MEPs were larger in middle-aged participants, whereas at high force levels (30% MVC) middle-aged participants had lower EMG values compared to young participants.

The EMG values in Hand-1 (both the 1-s as well as the 100-ms before the TMS stimulus data) showed a main effect of the force in Hand-1 [*F*_(1.2,21.9)_ = 312.18 and 284.70 for 1-s and 100 ms epochs, both *p* < 0.001; 5 versus 15 and 30% *p* < 0.001] and the force in Hand-2 [*F*_(3.4,62.1)_ = 7.51 and 6.51, both *p* < 0.001; 5% versus all force levels *p* < 0.05 for 1-s, 5% versus force >15% *p* < 0.05 for 100 ms epoch]. In addition, there was an Age-group by Hand-1 interaction [*F*_(1.2,21.9)_ = 5.19 and 4.65, both *p* < 0.03 for 1-s and 100-ms epochs] because the middle-aged compared with young subjects had lower EMG activity at 30% MVC (26.9 and 33.4% RMS-MVC; Figure 4D; only significant effect for 100 ms epoch). The EMG in Hand-2 was modulated with the force level of Hand-2 [*F*_(2.5,45.7)_ = 628.60, *p* < 0.001]. No further main or interaction effects occurred.

The MEPs were affected by both the force produced by Hand-1 [*F*_(1.7,30.6)_ = 74.09, *p* < 0.001] and the force produced by Hand-2 [*F*_(4.7,84.9)_ = 4.18, *p* = 0.002]. In addition, there was an Age-group by Hand-1 interaction [*F*_(1.7,30.64)_ = 5.29, *p* = 0.014]. In contrast to the RMS-EMG, the MEPs at 5% MVC were larger in the middle-aged subjects (30% of *M*_max_) than in young subjects (23% of *M*_max_; Figure 4B; 5 versus 15%, *p* = 0.006; 5 versus 30%, *p* = 0.02).

### Sustained contraction induced more associated activity in middle-aged subjects

#### Voluntary activity

**Figure 5:**
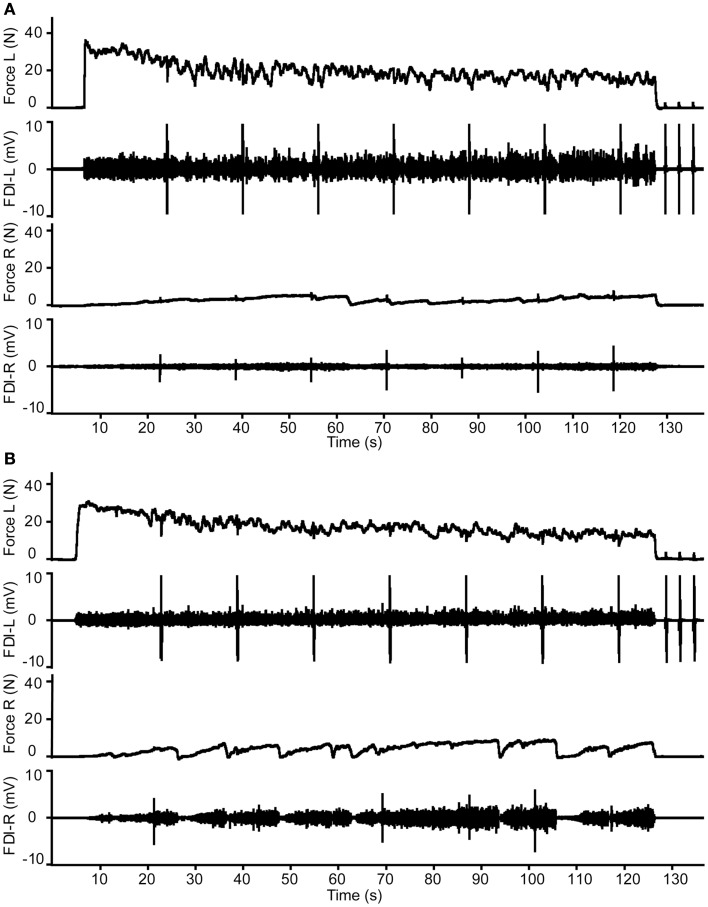
**Example of the changes in target and associated force and electromyography during the 2-min contraction with the FDI muscle of Hand-2 (left) in a young male (A) and middle-aged female participant (B)**. Please note the stronger activity in right hand (Hand-1) in the middle-aged participant.

**Figure 6:**
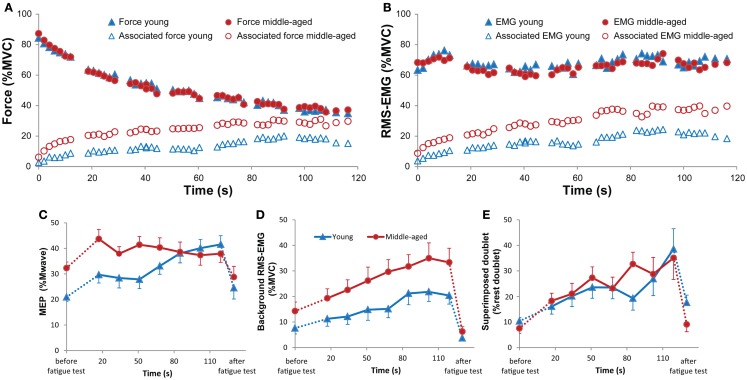
Changes in mean force [**(A)**, closed symbols] and RMS-EMG [2 s-values; **(B)**, closed symbols] expressed as % MVC, and mean associated force [**(A)**, open symbols] and RMS-EMG [2 s-values; **(B)**, open symbols] expressed as % MVC **(A,B)** during the sustained 2 min contraction for young (blue) and middle-aged subjects (red). During the test, stimuli are given to the cortex ipsilateral to the target muscle, and to the target (fatiguing) muscle. During the stimuli, no average force or RMS-EMG is calculated. Therefore, short time periods are missing in the graphs. **(C)** Changes in mean MEP amplitude (and standard error) during the sustained contraction for young (blue) and middle-aged (red) subjects separately. The first value shows the mean MEP during the contralateral MVC before the sustained contraction. The last values show MEP during the contralateral MVC after the sustained contraction. The analysis demonstrated a Time by Age group interaction (*p* = 0.006), revealing a decrease in MEP difference between the two Age groups with time. **(D)** Changes in mean (and standard error) RMS-EMG 100 ms before the TMS pulse. First and last values are the values obtained during the contralateral MVC. Note that the background RMS-EMG is associated activity. In contrast to the MEPs, the amount of ipsilateral activity increased progressively during the sustained contraction of young and middle-aged subjects (*p* < 0.001). **(E)** Changes in the mean (and standard error) superimposed doublet-force evoked in the FDI muscle that produces the maximal force during the sustained contraction. The doublet twitch increased throughout the sustained contraction reflecting a progressive decline in voluntary drive (*p* = 0.005), without a difference between the two Age-groups.

During the sustained contraction, the index finger abduction force progressively decreased from 86% MVC (7.0 SD) to 36% MVC (11 SD). The statistical analysis showed a significant decline over time [*F*_(45,765)_ = 196.27, *p* < 0.001; Figures 1D, 5, and 6A] but no Hand nor Age-group effects. The RMS-EMG values showed a more variable time course during the sustained task (Figure 6B), revealing a main effect of Time [*F*_(45,765)_ = 2.49, *p* < 0.001], and a Time by Hand interaction [*F*_(13.72,233.16)_ = 2.12, *p* = 0.012)]. The general trend was a decline in RMS-EMG in the left hand and a smaller change in the right hand.

During the sustained contraction, the FDI that performed the fatiguing task was stimulated seven times. The evoked force was used to estimate the voluntary drive during the sustained contraction. During the fatiguing task, the evoked doublet-force increased significantly (suggesting a decrease in voluntary drive) (*F* = 3.22, *p* = 0.005; Figure 6E); no main or interaction effects with Age-group were found.

#### Associated activity

During the sustained contraction, the amount of associated force and EMG increased significantly [force: *F*_(45,810)_ = 9.58, *p* < 0.001; RMS-EMG: *F*_(45,810)_ = 10.97, *p* < 0.001]. The amount of associated activity was significantly different between the Age-groups (Figures 5 and 6A,B). The associated force increased from 6.2 to 29.7% of MVC in the middle-aged subjects and from 2.7 to 15.2%MVC in the young subjects [Age-group: *F*_(1,18)_ = 7.05, *p* = 0.016]. The RMS-EMG showed a similar pattern; increase from 9.8 to 39.7% of MVC in the middle-aged subjects and from 3.8 to 18.4% of MVC for the young subjects [Age-group *F*_(1,18)_ = 5.99, *p* = 0.025].

#### Evoked potentials

During the sustained contraction, MEPs were evoked in the FDI showing the associated activity. Figure 6C shows the Time by Age-group interaction for the MEP amplitude [MEP peak: *F*_(5.2,94.4)_ = 3.53, *p* = 0.006; MEP area: *F*_(5.2,94.4)_ = 3.19, *p* = 0.009]. The first MEP during the sustained contraction increased in comparison with the control MVC before the sustained contraction (young: from 20.9 to 29.7% of *M*_max_, middle-aged: from 32.3 to 43.7% of *M*_max_). During the sustained contraction, the MEP in the middle-aged participants started at a higher value (44% of *M*_max_) than the MEP in the younger subjects and slowly decreased to 38% of *M*_max_, whereas in the young participants the MEP progressively increased (from 30 to 41% of *M*_max_) during the sustained contraction. As it was expected from the overall EMG measurement, the amount of background EMG upon which the MEP was evoked increased progressively during the fatiguing task [*F*_(8,144)_ = 12.45, *p* < 0.001; Figure 6D]. Overall, the EMG activity at the time of the TMS was higher in the middle-aged than younger subjects [*F*_(1,18)_ = 4.50, *p* = 0.06; Figures 6B,D]. Thus, the increase in MEP reflected the increase in background EMG in the young but not in the middle-aged subjects.

### Fatigue-related changes after the sustained contractions

The MVC of the fatigued FDI immediately after the fatiguing contraction decreased significantly to 67.2% of the pre-fatigue MVC [10.2 SD; *F*_(1,38)_, 43.40, *p* < 0.001], without an Age-group effect. The MVC of the non-fatigued FDI also showed a small decline in force to 92.2% of MVC (SD 10.5); this decline was larger in the right (88.1% of MVC, 10.4 SD) than in the left FDI (95.1% of MVC, 10.3 SD, *p* = 0.003); no main or interaction effects were found for Age-group. The doublet-force evoked in the fatigued FDI declined to 47.3% compared to the control values in both age groups (*p* = 0.22).

The amplitude and area of the MEPs evoked during contralateral MVCs did not change after the fatiguing task (106.7%, 38.2 SD of pre-fatigue values), whereas the background (associated) EMG was smaller but similar in the two age groups after the fatiguing contraction [pre-fatigue: 10.9% of RMS-MVC and post-fatigue: 4.9% of RMS-MVC; *F*_(1,12)_ = 12.41, *p* = 0.02].

There was a main effect of Time [*F*_(1,33)_ = 4.79, *p* = 0.05] for the doublet-force superimposed on the MVC before and after the fatiguing contraction. The evoked force was larger after the fatiguing contraction (pre-fatigue: 7.8% versus post-fatigue: 12.2% doublet force at rest), suggesting a decline in voluntary activation after the fatiguing task. No interaction effect with Age-group was found.

### Weak association between bilateral performance and associated activity

**Figure 7:**
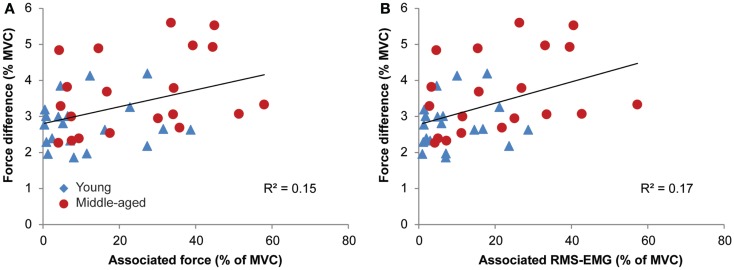
**Relationships between associated activity [EMG and force, in (A,B), respectively] and the measure of bilateral performance, i.e., force difference for young (blue triangle) and middle-aged subjects (red circles)**. Participants with high levels of associated activity tended to have an inferior bilateral performance.

The data demonstrated a weak but significant association between the measure of bilateral performance, i.e., force difference and the amount of associated activity during the MVCs for the left and right FDI (RMS-EMG: *R*^2^ = 0.17, *p* = 0.008; force: *R*^2^ = 0.15, *p* = 0.015; see Figure 7). Force difference was not significantly associated with the average amount of associated activity during the fatiguing sustained contraction (*p* > 0.5 for both average RMS-EMG and average force).

## Discussion

We compared the amount of associated activity and ipsilateral corticospinal excitability between young and middle-aged subjects during isometric contractions. We observed that the associated activity was ~2-fold higher in middle-aged (28% of MVC) when compared with young adults (11% of MVC) during brief as well as sustained MVCs (at 120 s: 15 versus 30% of MVC, for the young and middle-aged subjects). After carefully selecting MEPs that were not preceded by background EMG activity, no difference in ipsilateral corticospinal excitability was found between middle-aged versus young subjects during sub-maximal contractions. During the sustained contraction, MEPs were greater at the start of the contraction but there was no further increase *during* the contraction in middle-aged compared with young adults.

### Associated activity during brief maximal contractions is higher in middle-aged subjects

The increased levels of associated activity in middle-aged subjects during strong contractions confirm previous data obtained in elderly subjects [Bodwell et al., 2003 (65–85 years); Shinohara et al., 2003 (65–85 years); Baliz et al., 2005 (60–70 years); Addamo et al., 2009 (60–80 years); Fling and Seidler, 2012 (65–76 years)]. In a previous study, we demonstrated that the spinal motoneurons responsible for the associated contractions were activated via the contralateral corticospinal pathways; that is, the motor cortex ipsilateral to the target muscle (Zijdewind et al., 2006). Thus, our data demonstrate an additional increase in activity of these corticospinal pathways (ipsilateral to the target muscles) in middle-aged subjects during strong voluntary contractions. On basis of the present study, we cannot conclude whether these changes are present on cortical, spinal, or at both levels.

During a unilateral contraction, a MEP evoked in the ipsilateral motor cortex becomes facilitated (Hess et al., 1986; Ugawa et al., 1993; Stedman et al., 1998; Tinazzi and Zanette, 1998; Muellbacher et al., 2000; Liepert et al., 2001; Perez and Cohen, 2008, 2009). The larger MEPs during activation of the ipsilateral muscle are probably due to subliminal activation of the motor cortex contralateral to the target hemisphere. We did not find a significant difference in the ipsilateral MEPs between the two age groups during the sub-maximal contractions. It is possible that intra- and inter-cortical processes affect associated activity and ipsilateral excitability differently or we missed the increase in ipsilateral MEPs. The facilitation of the ipsilateral MEPs is more pronounced at high contralateral force levels (Hess et al., 1986; Ugawa et al., 1993; Stedman et al., 1998; Tinazzi and Zanette, 1998; Muellbacher et al., 2000; Liepert et al., 2001; Perez and Cohen, 2008, 2009) and since subjects were less able to prevent associated activation at higher force levels (see also Zijdewind and Kernell, 2001), hardly any MEPs at relatively high contralateral force levels remained.

Associated activity can be induced if the net excitation is above the spike-generating threshold for cortical neurons. Thus, the observation that middle-aged compared to young subjects more often show associated activity during strong contractions, suggests that the inhibition to the ipsilateral motor cortex is smaller or that the amount of excitation it receives is larger. It is often reported that intracortical inhibition (Peinemann et al., 2001; Hortobagyi et al., 2006; however, see Oliviero et al., 2006; Smith et al., 2009; McGinley et al., 2010), length of the silent period (Sale and Semmler, 2005) and interhemispheric inhibition (Talelli et al., 2008a,b; McGregor et al., 2011, 2013; Davidson and Tremblay, 2013) declines with age. However, one would expect to find an additional increase in ipsilateral MEPs (see Perez and Cohen, 2008). On the other hand, Perez and Cohen (2008) showed that the ipsilateral MEPs did correlate with changes in short-latency intracortical inhibition but less with changes in interhemispheric inhibition. So, maybe the increased associated activity during strong contractions does reflect differences in interhemispheric inhibition between the two age groups (Talelli et al., 2008a,b; Fling and Seidler, 2012). One other possible explanation could be a reduction in synchronized *I*-waves in middle-aged subjects (see also *larger MEPs during low contralateral activation*). A TMS stimulus can evoke both direct (*D*) and a series of indirect (*I*) waves (Kernell and Chien-Ping, 1967; Amassian et al., 1987). At low stimulation intensities, the contribution of *I*-waves to the excitation of corticospinal pathways is more important than the contribution from *D*-waves. A decrease in synchronized *I*-waves would thus result in smaller MEPs (see also discussion of Pitcher et al., 2003). Small changes in synchronization will probably have less effect during voluntary (or associated) activation. Hence, this could explain the higher level of associated activity during strong contractions without an increase in ipsilateral MEP size at rest.

The increase in associated activity in middle-aged subjects during effortful contractions is consistent with fMRI data that showed higher blood oxygenation level dependent (BOLD) activation of ipsilateral motor areas during motor tasks in old compared with young adults [Hutchinson et al., 2002 (54–76 years); Ward and Frackowiak, 2003 (26–80 years); Heuninckx et al., 2005 (62–71 years); Wu and Hallett, 2005 (57–73 years); Naccarato et al., 2006 (18–79 years); Riecker et al., 2006 (58–82 years); Talelli et al., 2008a (19–78 years); McGregor et al., 2011 (60–85 years)]. Yet, in most of these studies the authors claim that no associated activity was present. Still, the BOLD response in fMRI is more sensitive to a change in input, and processing of this input in a certain area, than to the actual output of this area (Logothetis, 2002) and thus subliminal activation of the ipsilateral primary motor cortex would show up during fMRI even if no associated muscle activity is observed. Only if the ipsilateral activity results in an increase in the corticofugal output that is strong enough to activate motoneurons, associated activity is seen. Nevertheless, even before associated movement is seen small motor unit potentials can be seen in the EMG resulting in a small change in EMG activity (often smaller than 25 μV). We expected that this increase in (subliminal) ipsilateral activation would show up as an increase in MEP. However after carefully selecting the MEPs without background EMG activity, we found no difference in MEP size between young and middle-aged subjects.

The relevance of the ipsilateral activation and the probably related associated activity is still unclear but better motor performance seemed to be associated with higher ipsilateral activation (Mattay et al., 2002). However, not all studies confirm the functional importance of the age-related increase in cortical activity. For example, Riecker et al. (2006) showed increased activation in elderly subjects (58–82 years) during a tapping task but with no further age-related increase in activity with increasing frequency. Additionally, recent data obtained in middle-aged (McGregor et al., 2011) and older subjects (McGregor et al., 2011) showed that increased iM1 activation was related to shorter ipsilateral silent periods and decreased motor function. On the other hand, in a recent study, Zimerman et al. (2012) demonstrated reduced motor learning in older subjects after cathodal DC-stimulation of the ipsilateral motor cortex. Cathodal DC-stimulation induces a reduction in excitability of the stimulated cortex (for reviews, see Nitsche and Paulus, 2011; Jacobson et al., 2012). Hence, their data suggest that the ipsilateral cortex is more important for motor learning in older compared with younger subjects (Zimerman et al., 2012). Our data showed a weak but significant association between the amount of associated activity and bimanual interference (as measured by the difference between target force and actual produced force), suggesting that high levels of associated activity could be detrimental for bimanual control (cf. McGregor et al., 2013). Again, the mechanisms underlying associated activity and ipsilateral activation could be different and probably, the relationship between these two variables is task-dependent.

### Associated activity during sustained contractions is higher in middle-aged subjects

The present study showed that, similarly to previous data in old adults [Shinohara et al., 2003 (66–80 years)], middle-aged compared with young subjects demonstrated higher associated activity already at the start of the voluntary muscle contraction. In the present experiment, this difference in associated activity was maintained throughout the sustained contraction and at the end of the 2-min contraction, the middle-aged compared with young subjects demonstrated a twofold larger level of associated activity. In the only other study that investigated associated activity during fatiguing contractions (Shinohara et al., 2003), the difference in associated activity between young and older subjects became progressively smaller as the contractions progressed. It is possible that this difference is due to age-differences; our middle-aged group was younger than the older subjects used by Shinohara et al. (2003) or to a difference in contraction type (anisometric contraction in Shinohara et al., 2003 versus isometric contractions in the present study).

During a sustained contraction, changes in excitability occur at different levels of the neuromuscular system. For instance, the excitability of the active motoneurons starts to decline as soon as 20 s after the start of the contraction (McNeil et al., 2009, 2011b). To maintain sufficient force production, the voluntary drive to motoneurons has to increase. This increase in drive is seen as an increase in activation of the contralateral primary motor cortex, as demonstrated by fMRI experiments (Dettmers et al., 1995; Liu et al., 2003; van Duinen et al., 2007; Post et al., 2009b). Furthermore, this increase in activity is accompanied by an increase in activity in the ipsilateral primary motor cortex (Dettmers et al., 1995; Liu et al., 2003; van Duinen et al., 2007; Post et al., 2009b), which could generate an increase in associated activity (see also Sehm et al., 2010). If the increase in voluntary drive is indeed reflected as an increase in associated activity, one would expect a progressive increase in associated activation during a sustained contraction. Since Shinohara et al. (2003) did not measure the voluntary drive, it is possible that during the sustained contraction the elderly subjects did not increase their drive similar to the young subjects and therefore did not increase their associated activity further as young subjects did. In the present experiment, both young and middle-aged subjects showed some decline in voluntary activation, as measured with the twitch superimposition technique, but we did not find a difference between the two age groups (see also Hunter et al., 2008).

### Age-related differences in ipsilateral corticospinal excitability during a sustained contraction

During a fatiguing task, the associated EMG progressively increases (Post et al., 2008). Despite the increase in background EMG in both groups, the MEPs remained unchanged in the middle-aged compared with young adults in whom the MEPs and the background EMG increased in parallel. The MEPs were comparable to the maximal MEPs obtained by Devanne et al., 1997; 45% *M*_max_) in the FDI muscle with different levels of background activity and stimulation intensity. The relationship between background EMG and MEP amplitude in the FDI muscle is not linear but the MEP peaks between 25 and 50% MVC (Devanne et al., 1997; Martin et al., 2006; Perez and Cohen, 2009). At higher background EMG levels, the MEP starts to decline. This reduction in MEP amplitude starts at relatively low force levels in the FDI muscle compared to for instance, the biceps brachii muscle (Martin et al., 2006). The main reason seems to be a change in the balance between two mechanisms of force control, recruitment gradation, and rate modulation. In the FDI muscle, only a few additional motor units become activated at force levels at or higher than 50% MVC, whereas in the biceps brachii muscle recruitment of new motor units continues up to 80% MVC (Milner-Brown et al., 1973; Kukulka and Clamann, 1981). Thus, at higher force levels, the FDI hand muscle completely relies on rate gradation.

After activation of a motoneuron, the motoneuron displays a refractory period followed by afterhyperpolarization (AHP). The time course of the AHP shows an exponential decline until the resting membrane potential (Matthews, 1996; Kernell, 2006). With increasing firing rates, the chance that a TMS pulse reaches a motoneuron during the refractory period or at the initial part of the AHP increases. During this period, the motoneuron is less likely to be activated by the TMS pulse. If smaller number of motoneurons are activated, this will result in a smaller TMS evoked muscle response. During the sustained contraction, the amount of associated activity increased from 20 to 35% of max RMS-EMG in the middle-aged subjects and 12–20% of max RMS-EMG. Thus, for the younger subjects the MEP still has more potential to grow during the sustained contraction, whereas in the middle-aged subjects the MEPs will be closer to their maximal value. Furthermore, there are indications that during a sustained (sub)maximal contraction the intrinsic properties of the motoneuron change to lower excitability levels (McNeil et al., 2011b). This would also result in smaller MEPs especially at higher firing rates. Thus, the lack of increase in MEPs in middle-aged subjects is probably due to fatigue-related changes on a spinal level, but these changes can be accompanied by time-related changes on cortical levels. In young subjects, the excitability changes at spinal levels are partly compensated by an increase in cortical activity, accompanied by increased ipsilateral activity (Post et al., 2008, 2009b). It is not known whether the increase in cortical activity is similar in young and middle-aged subjects but our data did not show any indication that the voluntary drive in the middle-aged subjects was smaller or the voluntary drive changed differently during the sustained contraction. Furthermore, the increase in the amount of associated activity suggests that also the ipsilateral motor cortex increases its activity with time.

### No difference in fatigue between young and middle-aged subjects

During the fatiguing maximal contraction, there were no age-related differences in the amount of fatigue as indexed by force loss, voluntary activation, and twitch force. Previous studies showed inconsistent data with respect to muscle fatigability of hand muscles in young and older subjects (see for review Allman and Rice, 2002). We are unaware of other studies reporting age-related differences in voluntary activation in the FDI but data in the upper arm muscles suggest that older subjects have a comparable but more variable voluntary activation [Hunter et al., 2008 (67–78 years)]. During their fatiguing task, the voluntary activation decreased as subjects repeated the 22-s-long MVCs. Both, their and our data showed a progressive but similar decline in voluntary activation in the two age groups (Hunter et al., 2008). The decline in voluntary activation was, however, larger than expected (Zijdewind et al., 1998). It is possible that the TMS-response in the ipsilateral motor cortex induced a short ipsilateral silent period resulting in a small decline in force 1.5 s before the muscle stimulation. We looked for ipsilateral silent periods but they were rarely visible (but cf. Fling and Seidler, 2012) and the duration of the reduced EMG was much shorter, but still the stimulation could have affected the attention of the subjects, resulting in a small extra decline in their voluntary activation. After the sustained contraction, the force decline in the fatigued and non-fatigued FDI (see also Post et al., 2008) was not different for the two groups.

### Greater MEPs at low forces during bilateral contractions in middle-aged subjects

At rest, there is evidence that with age the relationship between stimulus intensity and MEP size (the stimulus-response curve) shifts to the right without a change in the RMT (Pitcher et al., 2003). This observation demonstrates that with age, higher stimulus intensities are needed to obtain maximal MEPs. The most likely explanation for this shift in stimulus–response curve is reduced synchronization of *I*-waves (indirect activation of corticospinal neurons via interneurons) or changes in recruitment spacing in the motoneuron pool (e.g., due to loss of cortico-motoneurons, see Pitcher et al., 2003). In the present experiment, we used relatively low stimulus intensities, i.e., 110% RMT. It is therefore possible that with the TMS pulse we activated different portions of the corticospinal pathway in young or middle-aged subjects. However, we would expect to activate a smaller portion in the middle-aged subjects, resulting in smaller MEPs. At low force levels, the MEPs were larger in the middle-aged compared with young subjects (with similar background EMG). Although we only found a significant differential effect of background force and MEP size with age in the bilateral condition, the trend in the unilateral condition (*p* = 0.097) suggests that the effect was probably not confined to the bilateral condition.

The maximal rate at which motor units discharge action potentials declines with age (Kamen et al., 1995; Connelly et al., 1999). In hand muscles, all motor units are recruited at low force levels (below 50% MVC; Milner-Brown et al., 1973; Kukulka and Clamann, 1981). Thus, in hand muscles, high forces are extremely susceptible to changes in maximal firing rate and furthermore, these changes will affect the balance between recruitment and rate gradation. The change in balance between the two mechanisms of force control could be responsible for the larger MEP amplitude at low compared to high forces in middle-aged subjects. However, this possibility will require further confirmation in more detailed experiments.

In summary, our data show changes in associated activity during effortful contralateral contractions already in middle-aged subjects. Future experiments will have to determine if such changes in motor control at middle age are functionally relevant. Considering the widespread and increasing use of electronic devices that require high levels of bimanual coordination at work, the observed changes in corticospinal activity and bimanual control at middle age may have implications for screening individuals for such tasks and for designing interventions studies that modulate the levels of associated activity and improve motor function.

## Conflict of Interest Statement

The authors declare that the research was conducted in the absence of any commercial or financial relationships that could be construed as a potential conflict of interest.
